# Reprogramming of the developing heart by Hif1a-deficient sympathetic system and maternal diabetes exposure

**DOI:** 10.3389/fendo.2024.1344074

**Published:** 2024-03-05

**Authors:** Hana Kolesova, Petra Hrabalova, Romana Bohuslavova, Pavel Abaffy, Valeria Fabriciova, David Sedmera, Gabriela Pavlinkova

**Affiliations:** ^1^ Institute of Anatomy, First Faculty of Medicine, Charles University, Prague, Czechia; ^2^ Department of Developmental Cardiology, Institute of Physiology Czech Academy of Sciences (CAS), Prague, Czechia; ^3^ Laboratory of Molecular Pathogenetics, Institute of Biotechnology Czech Academy of Sciences (CAS), BIOCEV, Vestec, Czechia; ^4^ Faculty of Science, Charles University, Prague, Czechia; ^5^ Laboratory of Gene Expression, Institute of Biotechnology Czech Academy of Sciences (CAS), BIOCEV, Vestec, Czechia

**Keywords:** mouse model, maternal diabetes, coronary arteries, sympathetic neurons, cardiac sympathetic system

## Abstract

**Introduction:**

Maternal diabetes is a recognized risk factor for both short-term and long-term complications in offspring. Beyond the direct teratogenicity of maternal diabetes, the intrauterine environment can influence the offspring’s cardiovascular health. Abnormalities in the cardiac sympathetic system are implicated in conditions such as sudden infant death syndrome, cardiac arrhythmic death, heart failure, and certain congenital heart defects in children from diabetic pregnancies. However, the mechanisms by which maternal diabetes affects the development of the cardiac sympathetic system and, consequently, heightens health risks and predisposes to cardiovascular disease remain poorly understood.

**Methods and results:**

In the mouse model, we performed a comprehensive analysis of the combined impact of a *Hif1a*-deficient sympathetic system and the maternal diabetes environment on both heart development and the formation of the cardiac sympathetic system. The synergic negative effect of exposure to maternal diabetes and *Hif1a* deficiency resulted in the most pronounced deficit in cardiac sympathetic innervation and the development of the adrenal medulla. Abnormalities in the cardiac sympathetic system were accompanied by a smaller heart, reduced ventricular wall thickness, and dilated subepicardial veins and coronary arteries in the myocardium, along with anomalies in the branching and connections of the main coronary arteries. Transcriptional profiling by RNA sequencing (RNA-seq) revealed significant transcriptome changes in *Hif1a*-deficient sympathetic neurons, primarily associated with cell cycle regulation, proliferation, and mitosis, explaining the shrinkage of the sympathetic neuron population.

**Discussion:**

Our data demonstrate that a failure to adequately activate the HIF-1α regulatory pathway, particularly in the context of maternal diabetes, may contribute to abnormalities in the cardiac sympathetic system. In conclusion, our findings indicate that the interplay between deficiencies in the cardiac sympathetic system and subtle structural alternations in the vasculature, microvasculature, and myocardium during heart development not only increases the risk of cardiovascular disease but also diminishes the adaptability to the stress associated with the transition to extrauterine life, thus increasing the risk of neonatal death.

## 1 Introduction

The cardiac sympathetic system is a part of the autonomic nervous system that controls heart performance. This regulation is dependent on accurate connections between postganglionic sympathetic neurons and the heart, which is established during embryonic and postnatal development. Abolishing sympathetic system function affects survival due to cardiac failure (1–3). Most sympathetic postganglionic neurons innervating the heart are located in the stellate ganglion, with a smaller number present in the middle cervical and upper thoracic sympathetic ganglia of the sympathetic chains (4). Postganglionic neurons in these ganglia receive terminals from cardiac sympathetic preganglionic neurons in the upper thoracic spinal segments. Sympathetic innervation density is variable between regions both across and within the heart muscle layers and between the chambers of the heart (5). The highest density of sympathetic innervation is in the subepicardium and central conduction system, and it gradually decreases from the atria to the ventricles and from the base to the apex of the heart (5, 6). The cardiac sympathetic nervous system uses norepinephrine as a neurotransmitter.

Sympathetic neurons originate from neural crest cells that migrate near the dorsal aorta and form the primary sympathetic chain ganglia around embryonic day (E) 10.5 in mice (7, 8). These cells undergo neuronal and catecholaminergic differentiation, marked by the initiation of the expression of enzymes involved in norepinephrine biosynthesis, tyrosine hydroxylase (TH), and dopamine β-hydroxylase (7, 9). In the subsequent migration phase, sympathetic progenitors move away from the dorsal aorta to form secondary sympathetic ganglia at E13.5. Within these ganglia, sympathetic neuroblasts complete proliferation with a peak exit from the cell cycle at E14.5 and undergo final differentiation into sympathetic neurons (8). Proliferating sympathetic neurons begin to extend first axons as early as E12.5 (10, 11). Axons extend along the arteries attracted by vascular-derived guidance cues, including neurotrophin 3 (12), artemin (13), and endothelins (14). Nerve growth factor (NGF) produced by the heart controls the final stages of cardiac sympathetic innervation (15). Cardiomyocyte-derived Sema3a, a neural chemorepellent, is necessary for sympathetic innervation patterning by inhibiting axonal growth (16). The first axonal projections in the heart are detected in the dorsal subepicardium of the ventricles around E14 (17), and large coronary veins serve as an intermediate template for distal sympathetic axon extension (6). Subsequently, in the myocardial layer, sympathetic axons are guided by arteries toward the final target cells in the myocardium.

Target cell-derived factors control axon growth, branching, synaptic and electrophysiological properties, and release of neurotransmitters during development and in the establishment of mature properties of the sympathetic system (10, 18). Disrupting sympathetic innervation reciprocally affects heart development. For instance, neonatal chemical sympathectomy disrupts the cardiomyocyte cell cycle, resulting in a reduced heart size (19). The treatment by β-adrenergic receptor (AR) antagonists, which are used clinically to treat conditions associated with excessive effects of norepinephrine, promoted the progression of cytokinesis in neonatal mice, which reduced adverse remodeling after myocardial infarction in adults (20). Similarly, infusions of β-AR blockers induce significant neurite outgrowth in the *in vitro* assay system of neonatal sympathetic neurons and myocardial sympathetic axon density in the rat heart with elevated ventricular contractility (21). Sympathetic neurons have a significant role in the regulation of cardiomyocyte maturation, as shown by *in vitro* co-cultures of human induced pluripotent stem cell (hiPSC)-derived cardiomyocytes with sympathetic neurons (22). Furthermore, sympathetic defects have been associated with sudden infant death syndrome, cardiac arrhythmic death, and certain congenital heart defects in children (23, 24).

Changes in cardiac sympathetic innervation and sympathetic system activity are implicated in many pathologies in adults, including sudden cardiac death, myocardial ischemia, cardiac death, hypertension, diabetic heart disease, and heart failure (25, 26). Emerging evidence suggests that many of the sympathetic dysfunctions in various pathological heart conditions have a developmental origin (19, 20, 27, 28). Using conditional deletion of oxygen-sensitive subunit HIF-1α, we previously revealed a key role for the transcription factor hypoxia-inducible factor 1 (HIF-1) in the development of sympathetic neurons and sympathetic innervation of the developing heart and its negative effects on heart function in adults (29). HIF-1 coordinately regulates responses to hypoxia and ischemia and plays multifactorial roles in pathophysiological responses in myocardial ischemia, infarction, metabolic and structural remodeling, and heart failure (30–33). Additionally, HIF-1-regulated pathways direct cardiac development (34–36). A number of studies highlight the combinatorial effects of HIF-1 deregulation and environment on heart development, including fetal hypoxia or maternal diabetes, influencing the cardiovascular and metabolic health of offspring (37–40). Despite significant progress in understanding this phenomenon known as fetal or developmental programming [reviewed in (41–43)], numerous questions pertaining to its underlying penetrance and disease predisposition remain unresolved.

In this study, we present a comprehensive analysis of the combined impact of the *Hif1a*-deficient cardiac sympathetic system and the adverse maternal diabetes environment on embryonal development. Our analysis of *Hif1aCKO* embryos revealed a negative synergistic effect of *Hif1a* deletion and the diabetic environment on the development of cardiac innervation and chromaffin cells of the adrenal medulla of the sympathetic system, affecting heart development. Thus, exposure to maternal diabetes and *Hif1a*-deficient cardiac sympathetic system heighten the risk of cardiovascular disease in the offspring.

## 2 Materials and methods

### 2.1 Experimental animal models

This study was approved by the local Animal Care and Use Committee of the Institute of Molecular Genetics CAS and the Institute of Anatomy, First Faculty of Medicine, Charles University. All experiments were performed with embryo littermates (females and males). Animals were housed in a controlled environment with 12-h light/dark cycles and free access to water and food.

The previously described experimental model of the conditional deletion of *Hif1a* (*Hif1aCKO*) genotype *Isl1^tm1(cre)Sev^/^+^;Hif1a^loxP/loxP^* was used (29). Briefly, floxed *Hif1^atm3Rsjo^* with exon 2 of the *Hif1a* gene flanked by loxP sites (44) on a mixed C57BL/6J;C57BL/6N genetic background were obtained from Jackson Laboratories (Bar Harbor, ME, USA; #Strain 007561). *Isl1^Cre/+^* mice were on the FVB background. *Hif1a^loxP/+^* or *Hif1a^loxP/loxP^* mice without the *Isl1-Cre* allele individuals were used as the control. For the breeding scheme, female mice *Hif1a^loxP/loxP^* were crossed with *Hif1a^loxP/+;^ Isl1^Cre/+^* males, in which the *Isl1-Cre* knock-in allele was inherited paternally to minimize the potential influence of maternal genotype on the developing embryos.

To visualize sympathetic neurons, we used *tdTomatoAi14* reporter mice with *Rosa-CAG-LSL-tdTomato* allele (Ai14, B6.Cg-*Gt(ROSA)26Sor^tm14(CAG-tdTomato)Hze^*, Stock No. 7914 Jackson Laboratories) to generate the reporter *Hif1aCKO*-*Ai14* (genotype: *Hif1a^loxP/loxP^;Isl1^Cre^;tdTomatoAi14*) and control-*Ai14* mice (genotype: *Hif1a^loxP/+^;Isl1^Cre^;TdtomatoAi14*). To examine the formation of the secondary sympathetic chain, we used *peripherin-enhanced green fluorescent protein* (*Prph-eGFP*) genomic reporter transgenic mice (45, 46) and crossed them with the *Hif1a* mutant line.

To visualize the pattern of the developing coronary arteries, *Hif1a^loxP/loxP^* mice were crossed *to Cx40:eGFP* strain with eGFP signal in the coronary arteries, the atria, the atrioventricular node, and the His–Purkinje system (47). Double homozygote females (*Cx40:^eGFP/+^*;*Hif1a^loxP/loxP^*) with background CD1/129SvJxSwiss were then crossed with *Hif1a^loxP/+^;Isl1^Cre^* males.

**Figure 1:**
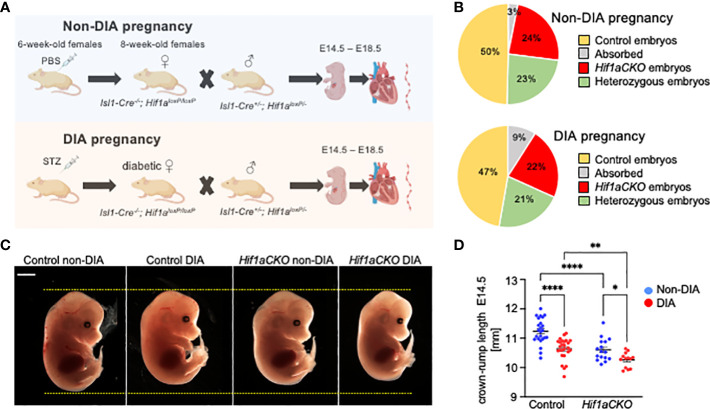
Maternal diabetes affects the distribution of genotypes and size of embryos. **(A)** Experimental design. Diabetes was induced in 6-week-old females by intraperitoneal injections of streptozotocin (STZ). Diabetic females were mated with males, and embryos were collected for analysis from E14.5 to E18.5 from diabetic and non-diabetic pregnancies. Created with BioRender.com. **(B)** The distribution of embryo genotypes collected at E14.5 was influenced by maternal diabetes. χ^2^ test **p = 0.0012, non-diabetic pregnancies: n = 33 absorbed, n = 260 *Hif1aCKO*, n = 253 heterozygous *Hif1aCKO*, and n = 538 control embryos from 124 litters; diabetic pregnancies: n = 18 absorbed, n = 45 *Hif1aCKO*, n = 42 heterozygous *Hif1aCKO*, and n = 94 control from 22 litters. **(C)** Representative images of E14.5 embryos. Scale bar, 2 mm. **(D)** The evaluation of the crown–rump length of control and mutant embryos from non-diabetic and diabetic pregnancies at E14.5. Data are presented as mean ± SEM, n = 24 non-DIA Control, n = 26 DIA Control, n = 16 non-DIA *Hif1aCKO*, and n = 13 DIA *Hif1aCKO*. Two-way ANOVA followed by *post hoc* Fisher’s multiple comparisons test; *p < 0.05, **p < 0.01, ****p < 0.0001.

The noon of the day on which the vaginal plug was found was designated E0.5. Animals were euthanized by cervical dislocation in our study. Embryos were collected for analysis at different ages (**Figure 1A**). All comparisons were made between animals with the same genetic background. Phenotyping and data analysis were performed blind to the genotype of the mice. Genotyping was performed by PCR on tail DNA (**Supplementary Table 1**).

### 2.2 Diabetes induction

Diabetes was induced in 6-week-old females (*Hif1a^loxP/loxP^*) by two intraperitoneal injections of 100 mg/kg body weight of streptozotocin (STZ, S0130, Sigma-Aldrich, Dorset, UK) within a 1-week interval, as described (37, 48). The level of blood glucose was checked in a drop of blood from the tail vessel using a glucometer (CONTOUR plus ONE, Ascensia Diabetes Care, Basel, Switzerland). Mice with a level of glucose maintained above 13.9 mmol/l in blood were classified as diabetic. Diabetic females were mated with males, and the next morning, the vaginal plug was checked. Maternal blood glucose levels at the time of embryo collection are shown in **Supplementary Figure 1**. Embryos collected from diabetic pregnancies were labeled as "diabetic" or "DIA", and from non-diabetic pregnancies were labeled as "non-diabetic" or "non-DIA".

### 2.3 Immunohistochemistry and morphological evaluations

Whole-mount immunohistochemical staining of embryonic hearts (E16.5 and E18.5) was performed on cleared tissue, as described (49). Embryos were perfused with 0.05% heparin in phosphate-buffered saline (PBS) and fixed in 4% paraformaldehyde (PFA) for 90 minutes. The heart was cleared using CUBIC reagent at 37°C with gentle shaking for 1 week (50). Used primary and secondary antibodies are described in **Supplementary Tables 2** and **3**. The nuclei were stained with Hoechst. The fluorescent signals were detected using LSM 880 NLO (AxioObserver Z1, Carl Zeiss, Oberkochen, Germany) and AxioZoomV16 (Carl Zeiss, Germany).

Positive areas (TH and TUJ1) were quantified using the Threshold tool in NIH ImageJ software and expressed as a percentage of total areas (heart and adrenal gland). The total number of positive cells (NeuN) in the whole stellate ganglion (STG) was quantified using a cell counter in NIH ImageJ software. Microvasculature density was assessed using immunohistochemically stained 8-µm paraffin sections labeled with anti-PECAM-1 (an endothelial marker) and wheat germ agglutinin (WGA). Sections were imaged on an Olympus confocal microscope, and positive signals of PECAM-1 and WGA were combined and thresholded in NIH ImageJ. Then, the positive area was measured in the region of interest (compact layer) in the right ventricle. Subsequently, the measured microvasculature-positive area was normalized to the background signal (all tissue autofluorescence) and expressed as a percentage, thus leading to the density of microvasculature in the right ventricular wall.

To visualize the pattern of the developing coronary arteries together with TH^+^ innervation, the hearts were dissected and immunolabeled with anti-TH and Cy5-conjugated secondary antibodies. The atria were then carefully cut off to expose the origin of the coronary arteries at the base of the heart, cleared in CUBIC for 48 h (51), and visualized with 4× and 10× dry objectives on an Olympus FluoView 1000 confocal system. For quantification of TH^+^ innervation, maximum intensity projections (MIPs) of confocal series taken with a 4× objective with 25-μm z-step were used. The image from the far-red (Cy5) channel containing the signal from the anti-TH antibody was oriented with the base on the top and the apex at the bottom (Valentine projection). Two semicircular segmented lines at 25% and (basal) and 75% (apical) apex–base distance were drawn in ImageJ, and line profiles were then generated (**Supplementary Figure 2**). The peaks in fluorescence intensity, corresponding to individual nerve bundles, were then easily counted in an unbiased manner.

### 2.4 Light-sheet fluorescence microscopy and analysis of images

Embryonic hearts were microdissected from non-diabetic and diabetic control and *Hif1aCKO* embryos (E16.5 and E18.5). An advanced CUBIC protocol (49) with some modifications (50) was used for tissue clearing to enable efficient imaging by light-sheet microscopy. Whole-mount immunohistochemical staining of embryonic hearts was performed, and samples were stored before imaging in Cubic 2 at room temperature. The secondary sympathetic chain was microdissected from non-diabetic and diabetic control-*Ai14* and *Hif1aCKO-Ai14* embryos (E14.5) and whole-mount stained with NeuN. Zeiss Lightsheet Z.1 microscope with illumination objective Lightsheet Z.1 5×/0.1 and detection objective Dry objective Lightsheet Z.1 5×/0.16 was used for imaging at the Light Microscopy Core Facility of the Institute of Molecular Genetics of the Czech Academy of Sciences. IMARIS software v8.1.1 (Bitplane AG, CA, San Francisco, USA) was used for image processing.

### 2.5 RNA sequencing of fluorescence-activated cell-sorted sympathetic ganglion neurons

Sympathetic ganglia were dissected from E14.5 embryos *Hif1aCKO-Ai14;Prph-eGFP* (n = 3) and *Control-Ai14;Prph-eGFP* (n = 2). Sympathetic chains were homogenized, and neuronal cells were dissociated using 0.1% collagenase (C9263, Sigma-Aldrich, UK) and 0.05% trypsin in Dulbecco’s PBS for 7 minutes (T4799, Sigma-Aldrich, UK). Enzymatic activity was stopped by adding fluorescence-activated cell sorting (FACS) buffer [2% fetal bovine serum (FBS) in Dulbecco’s PBS and 10 mM EGTA]. FACS of eGFP and tdTomato-positive cells was performed at the Imaging Methods Core Facility at BIOCEV on a BD FACS Aria Fusion flow cytometer operated using BD FACSDiva™ Software. A total of 100 eGFP^+^ and tdTomato^+^ cells per biological sample were collected into individual wells of a 96-well plate containing 5 µL of lysis buffer of NEBNext single-cell low input RNA library prep kit for Illumina (#E6420, New England Biolabs, Ipswich, MA, USA). Plates were frozen immediately on dry ice and stored at −80°C. The total time from euthanasia to cell collection was ∼3 h.

The RNA library preparation, RNA sequencing (RNA-seq), and data processing were performed as described previously (27). Briefly, the NEBNext single-cell low-input RNA library prep kit for Illumina (#E6420, New England Biolabs) was used for library generation at the Gene Core Facility (Institute of Biotechnology CAS, Czechia), and the libraries were sequenced on an Illumina NextSeq 500 next-generation sequencer with NextSeq 500/550 High Output kit 75 cycles (Illumina #200024906) at the Genomics and Bioinformatics Core Facility (Institute of Molecular Genetics CAS, Czechia). RNA-seq reads in FASTQ files were mapped to the mouse genome GRCm38 primary assembly release M8 using STAR [version 2.7.0c (52)]. Using cutadapt v1.18 (53), the number of reads (minimum, 32 million; maximum, 73 million) was trimmed by Illumina sequencing adaptor and bases with reading quality lower than 20; subsequently, reads shorter than 20 bp were filtered out. TrimmomaticPE version 0.36 (54). Ribosomal RNA and reads mapping to UniVec database were filtered out using bowtie v1.2.2. with parameters -S -n 1 and SortMeRNA (55). A count table was generated using the Rsubread v2.0.1 package with default parameters without counting multi-mapping reads. The raw RNA-seq data were deposited at NIH GEO under accession number GSE250606 (https://www.ncbi.nlm.nih.gov/geo/query/acc.cgi?acc=GSE250606).

DESeq2 [v1.26.0 (56)] default parameters were used to normalize data and compare the different groups. Differentially expressed genes between *Hif1aCKO* and control sympathetic neurons were identified based on an adjusted p-value p_adj_ < 0.05, log2 fold change (log2FC >0.3, <−0.3), and a base mean ≥50. The functional annotation of the differentially expressed genes was performed using g: Profiler (Raudvere et al., 2019). Complete query details are available in Query info Tables in Dataset S1. The resulting GEM and combined GMT files using term size <1,800 were loaded into Cytoscape (57) plugin “EnrichmentMap” (58) using 0.01 false discovery rate (FDR) q-value cutoff to generate a network. The edge cutoff was set to 0.35, and nodes were set to 0.007 Q value.

### 2.6 Quantitative real-time PCR

RNA was isolated from the microdissected sympathetic chains of individual embryos from diabetic and non-diabetic litters using TRIzol (Invitrogen, Carlsbad, CA, USA). The concentration and purity were quantified using NanoDrop (ND-2000 Spectrophotometers, Thermo Fisher Scientific, Waltham, MA, USA). cDNA samples were prepared using Maxima H Minus First Strand cDNA Synthesis Kit with dsDNA (#K1682, Thermo Scientific, USA) from 300 ng isolated RNA/sample. Quantitative real-time PCR (qRT-PCR) was performed using 10× diluted cDNA samples. cDNA at a volume of 4 µL was added to 5 µL of SybrGreen (GrandMaster Mix, TATAA Biocenter, Gothenburg, Sweden) with 0.2 µM reverse and forward primers. Primers were designed using Primer3 software, and sequences are shown in **Supplementary Table S2**. Validation of RNA-seq targets was performed using Bio-Rad C1000 Thermal Cycler (CFX384 Real-Time System, Bio-Rad Laboratories, Hercules, CA, USA), and activation was performed using AmpliTaq at 95°C for 10 minutes, followed by 40 cycles at 95°C for 15 s for denaturation and 60°C for 60 s for extension. The relative expression levels of mRNA of target genes were normalized to the reference gene *Hprt1*. All reactions were conducted in duplicates, and the data were calculated using the ΔΔCp method, as previously described (37, 59). Primer sequences are presented in **Supplementary Table 4**.

### 2.7 Statistical analysis

Statistical analyses were performed using two-way ANOVA (GraphPad Prism 10), testing differences among experimental groups based on the genotype and experimental condition (diabetic or non-diabetic pregnancy) followed by multiple Fisher’s comparisons; results are expressed as mean ± SD or mean ± SEM, with significance level p < 0.05. A chi-square (χ^2^) test was used to compare the distribution of genotypes between the non-diabetic and diabetic groups (GraphPad Prism 10). Sample sizes and individual statistical results for all analyses are provided in the figure legends and tables.

## 3 Results

### 3.1 Maternal diabetes affects genotype distribution and the size of embryos

We analyzed the combined impact of the *Hif1a*-deficient cardiac sympathetic system and the adverse maternal diabetes environment on heart development (schematics of experimental study design in **Figure 1A**). The observed distribution of embryo genotypes collected at E14.5 was influenced by maternal diabetes (**p = 0.0012, χ^2^ test; **Figure 1B**). We did not observe any developmental delay or structural abnormalities among embryos from diabetic and non-diabetic pregnancies. However, we identified a significant decrease in the average crown–rump length of both diabetic control and *Hif1aCKO* embryos compared to non-diabetic controls at E14.5 (**Figures 1C, D**). Furthermore, the length of diabetic *Hif1aCKO* embryos was significantly smaller than that of diabetic controls or non-diabetic *Hif1aCKO*, indicating a synergistic effect of *Hif1a* deletion and the diabetic environment.

### 3.2 Cardiac sympathetic innervation is attenuated by *Hif1a* mutation and diabetic exposure

**Figure 2:**
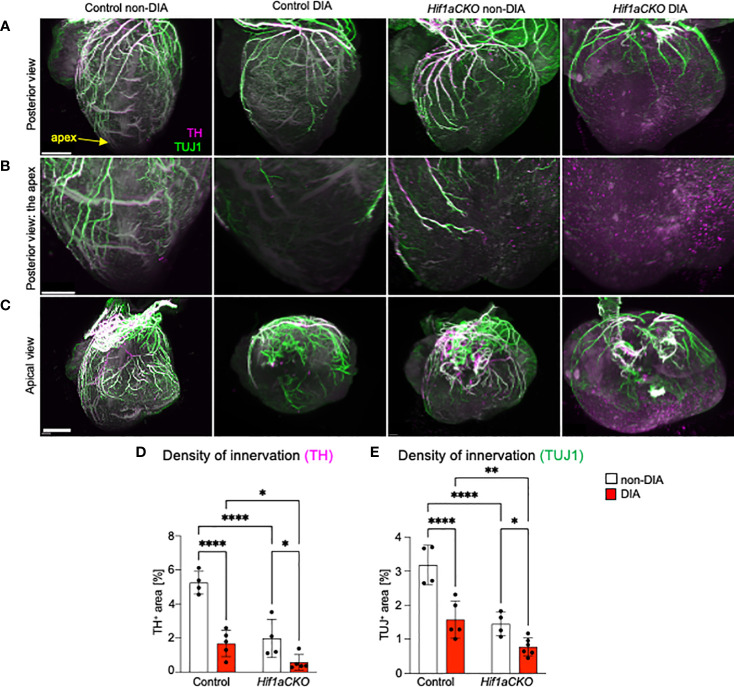
Reduced innervation in the diabetic *Hif1aCKO* heart at E16.5. **(A)** Representative images of immunolabeling of sympathetic innervation using anti-tyrosine hydroxylase (TH) and anti-class III β-tubulin (TUJ1) in the heart in the posterior view (scale bars, 500 µm). See also **Supplementary Videos S1**-**S4**. **(B)** The heart apex in detail in the posterior view (scale bar, 300 µm) and **(C)** the apical view (scale bar, 600 µm). **(D)** TH^+^ and **(E)** TUJ1^+^ fibers were quantified using the threshold tool in ImageJ and expressed as a percentage of the measured heart area. Cardiac innervation is reduced in *Hif1aCKO*, and the effect is further potentiated by diabetes. Data are presented as the mean ± SD (n = 4–5 samples). Two-way ANOVA followed by *post hoc* Fisher’s multiple comparisons test; *p < 0.05, **p < 0.01, ****p < 0.0001.

To assess the extent of cardiac innervation, we employed double immunolabeling of TH, a marker of sympathetic neurons and sympathetic innervation, and class III β-tubulin (TUJ1), a neuronal marker expressed in all cardiac fibers, representing sympathetic, parasympathetic, and sensory innervation. While parasympathetic cardiac innervation precedes sympathetic innervation, sympathetic fibers move alongside established vagal nerve tracts to innervate the heart (60). In the developing mouse heart, autonomic innervation precedes sensory innervation, with sensory axons becoming detectable at E18.5 (6, 61). In line with our previous study (29), the conditional deletion of *Hif1a* in sympathoadrenal progenitor lineage led to a profound deficit in cardiac sympathetic innervation (**Figures 2A–D**; **Supplementary Video S1**-**S4**). At E16.5, our immunohistochemical staining revealed that the majority of TH^+^ axons in the posterior part of the ventricles of diabetic *Hif1aCKO* were lost, with no cardiac fibers observed in the apex (**Figures 2B, D**). Maternal diabetes and *Hif1a* mutation significantly reduced TH^+^ and TUJ1^+^ cardiac innervation when compared to hearts from non-diabetic pregnancies and control hearts, respectively (**Figures 2D, E**). Notably, a negative synergistic effect of *Hif1a* deletion and the diabetic environment on cardiac innervation was observed when comparing *Hif1aCKO* and control hearts from diabetic pregnancies.

**Figure 3:**
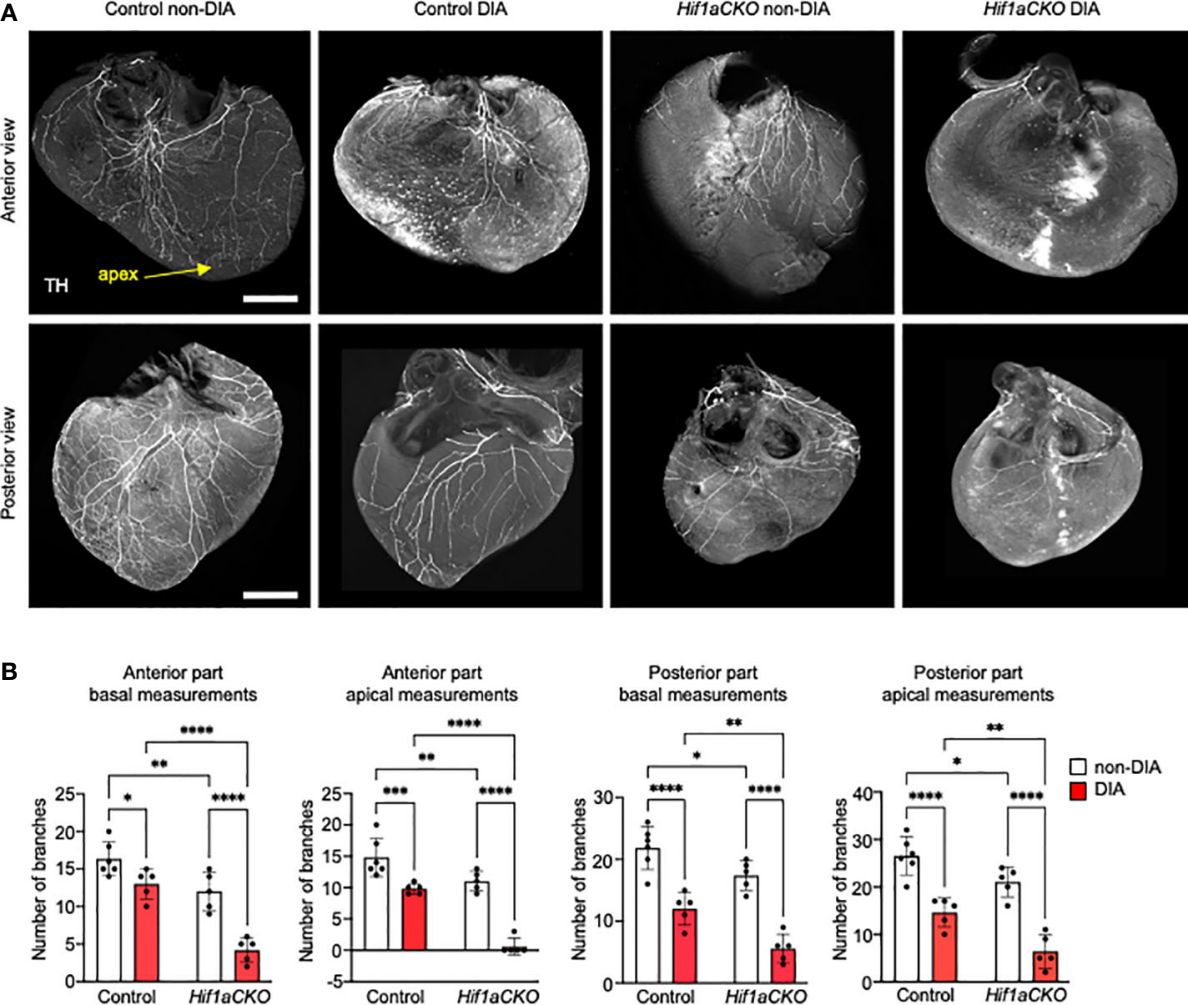
Sympathetic innervation of the anterior and posterior parts of the ventricles reduced by *Hif1a* deletion and maternal diabetes at E17.5. **(A)** Representative images of immunohistochemical staining of sympathetic innervation in the anterior and posterior parts of the ventricles using tyrosine hydroxylase (TH) (scale bar, 500 µm). Posterior part of heart ventricles contains more branches, and their plexus is more complex compared to the anterior part of heart ventricles. The greatest reduction of sympathetic innervation is noticeable in diabetic *Hif1aCKO* with a few main branches innervating the anterior part of the ventricular wall, but all distal branches of the ventricles are lost. Similarly, the posterior part of diabetic *Hif1aCKO* ventricles has a significant loss of innervation with only few remaining nerves. **(B)** The fluorescence intensity corresponding to individual nerve proximal and distal branches in the ventricular wall was quantified using ImageJ at 25% (basal) and 75% (apical) apex–base distance (**Supplementary Figure S2**). Data are presented as the mean ± SD (n = 5–6 samples). Two-way ANOVA followed by *post hoc* Fisher’s multiple comparisons test; *p < 0.05, **p < 0.01, ***p < 0.001, ****p < 0.0001.

Next, we evaluated the sympathetic innervation of the posterior and anterior parts of the ventricular wall in detail. Sympathetic innervation, as marked by anti-TH, was notably reduced in the *Hif1aCKO* ventricles, with a loss of both proximal and distal branches compared to the control hearts at E17.5, mirroring the observation at E16.5 (**Figure 3A**). The difference at the base of the heart, where the thick bundles dominated, was less affected between genotypes; however, a clear decrease in the number of (usually thinner) bundles was evident at the apex, attesting to a deficient innervation. The maternal diabetes exposure resulted in a significant attenuation of sympathetic innervation in the control hearts (**Figures 3A, B**). However, the synergistic detrimental effects of *Hif1a* deletion and diabetic pregnancy were most pronounced in the diabetic *Hif1aCKO* hearts. Only a few main branches innervated the basal area of the myocardial wall in the anterior view, and all distal ventricular branches were missing. While the posterior part of the heart contained a higher number of branches with a more complex plexus, the combination of *Hif1a* deletion and maternal diabetes led to a nearly complete loss of innervation, with only a few remaining nerves (**Figures 3A, B**).

**Figure 4:**
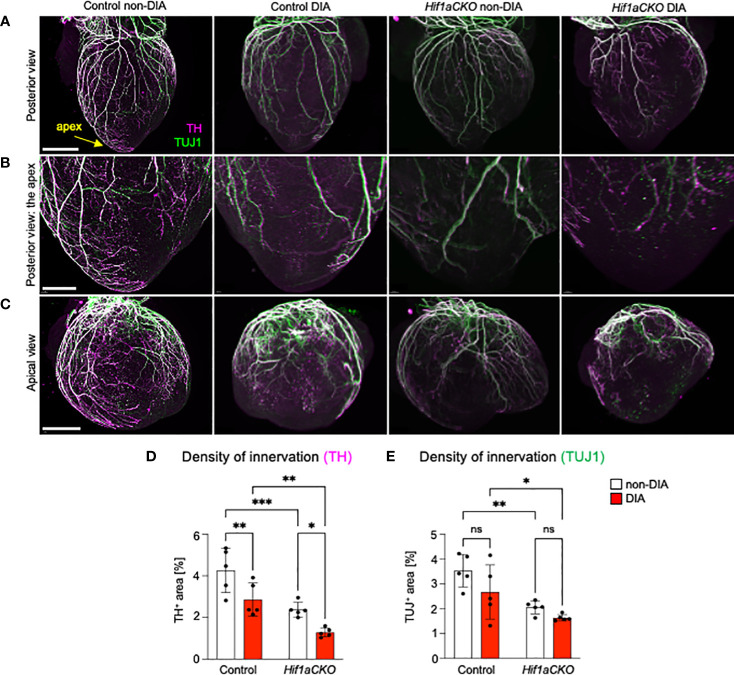
Reduced sympathetic innervation in the diabetic *Hif1aCKO* heart at E18.5. **(A)** Representative images of immunohistochemical staining of sympathetic innervation using tyrosine hydroxylase (TH) and class III β-tubulin (TUJ1) in the posterior view of the heart (scale bar, 1,000 µm), **(B)** in the apex in detail (scale bar, 500 µm), and **(C)** in detail the apical view of the heart (scale bar, 1,000 µm). **(D)** TH^+^ and **(E)** TUJ1^+^ innervations were quantified using the threshold tool in ImageJ and expressed as a percentage of the measured heart area. The combination of *Hif1a* deficiency and exposure to maternal diabetes led to the greatest reduction in sympathetic innervation. Data are presented as the mean ± SD (n = 5). Two-way ANOVA followed by *post hoc* Fisher’s multiple comparisons test; *p < 0.05, **p < 0.01, ***p = 0.0002. ns, not significant.

At E18.5, the most pronounced deficit in cardiac sympathetic innervation was detected in *Hif1aCKO* embryos exposed to maternal diabetes, indicating a long-lasting synergistic effect of *Hif1a* deletion and the diabetic environment (**Figures 4A–D**). Conversely, TUJ1^+^ axons were equally reduced in both non-diabetic and diabetic *Hif1aCKO* hearts, highlighting the impact of *Hif1a* mutation rather than maternal diabetes (**Figure 4E**). Similarly, exposure to maternal diabetes did not significantly affect TUJ1 innervation in the control heart compared to control hearts from non-diabetic embryos. Both sympathetic and TUJ1^+^ fibers were significantly reduced in non-diabetic *Hif1aCKO* hearts when compared to non-diabetic control hearts (**Figures 4D, E**), indicating abnormalities in the formation of cardiac innervation of *Hif1aCKO*. Consequently, these changes could have significant implications for heart function and postnatal survival of *Hif1aCKO* mutants.

### 3.3 Myocardial changes induced by maternal diabetes

**Figure 5:**
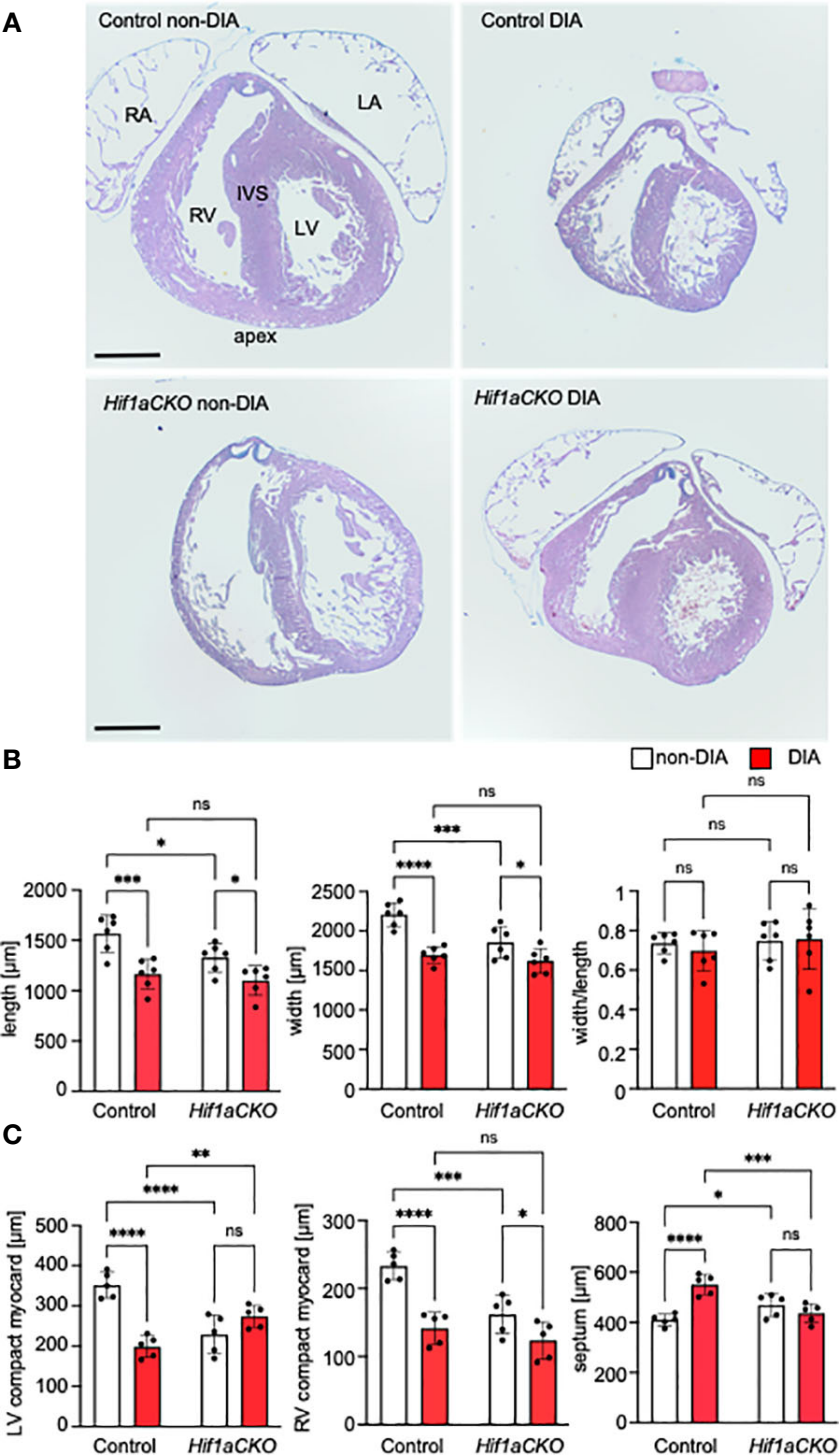
Morphological changes induced by the *Hif1a*-deficient sympathetic system and maternal diabetes exposure. **(A)** Representative images of histological staining with Alcian Blue/hematoxylin and eosin show transverse sections of E17.5 hearts; four-chamber view at the level of the pulmonary valve. RV, right ventricle; LV, left ventricle; IVS, interventricular septum; RA, right atrium; LA, left atrium. **(B)** The length and width of the mutant heart were significantly decreased; this effect was also induced by exposure to maternal diabetes. The heart shape, as assessed by the width/length ratio, remained similar among groups. **(C)** The ventricular thickness of the compact myocardium layer (μm) was reduced in the *Hif1aCKO* and diabetic hearts compared to controls. In contrast, the interventricular septum was thickened in the normoglycemic mutants versus controls and in the control group with diabetes. Data are presented as the mean ± SD (n = 5). Two-way ANOVA followed by *post hoc* Fisher’s multiple comparisons test, *p < 0.05, **p < 0.01, ***p < 0.001, ****p < 0.0001. ns, not significant. Scale bars, 500 µm.

Given the implication of the sympathetic nervous system in heart size regulation and cardiomyocyte proliferation (19, 20), we conducted an analysis of the wall thickness of both the left ventricle (LV) and right ventricle (RV) and the thickness of the interventricular septum, using the sections of the E17.5 heart (**Figure 5A**). First, the length of the heart (from apex to base) and width across the widest part of both ventricles were measured. Maternal diabetes and *Hif1a* mutation significantly reduced both measured parameters, although the ratio of length and width was not altered, indicating that the ventricular proportions were similar among groups (**Figures 5A, B**). The compact myocardium of the LV and RV of the *Hif1aCKO* heart was significantly thinner compared to those of their control littermates from non-diabetic pregnancies (**Figures 5A, C**). Additionally, the control embryos from the diabetic pregnancy exhibited thinner LV and RV walls when compared to control non-diabetic embryos. Interestingly, the thickness of the interventricular septum increased in both diabetic and non-diabetic *Hif1aCKO* and diabetic control hearts compared to the control heart of embryos from non-diabetic pregnancies (**Figures 5A, C**). However, we did not detect an additive effect of the combination of *Hif1a* mutation and the diabetic environment on these parameters when compared to diabetic control embryos.

### 3.4 Diabetes and *Hif1a* deletion result in abnormalities in coronary vasculature

**Figure 6:**
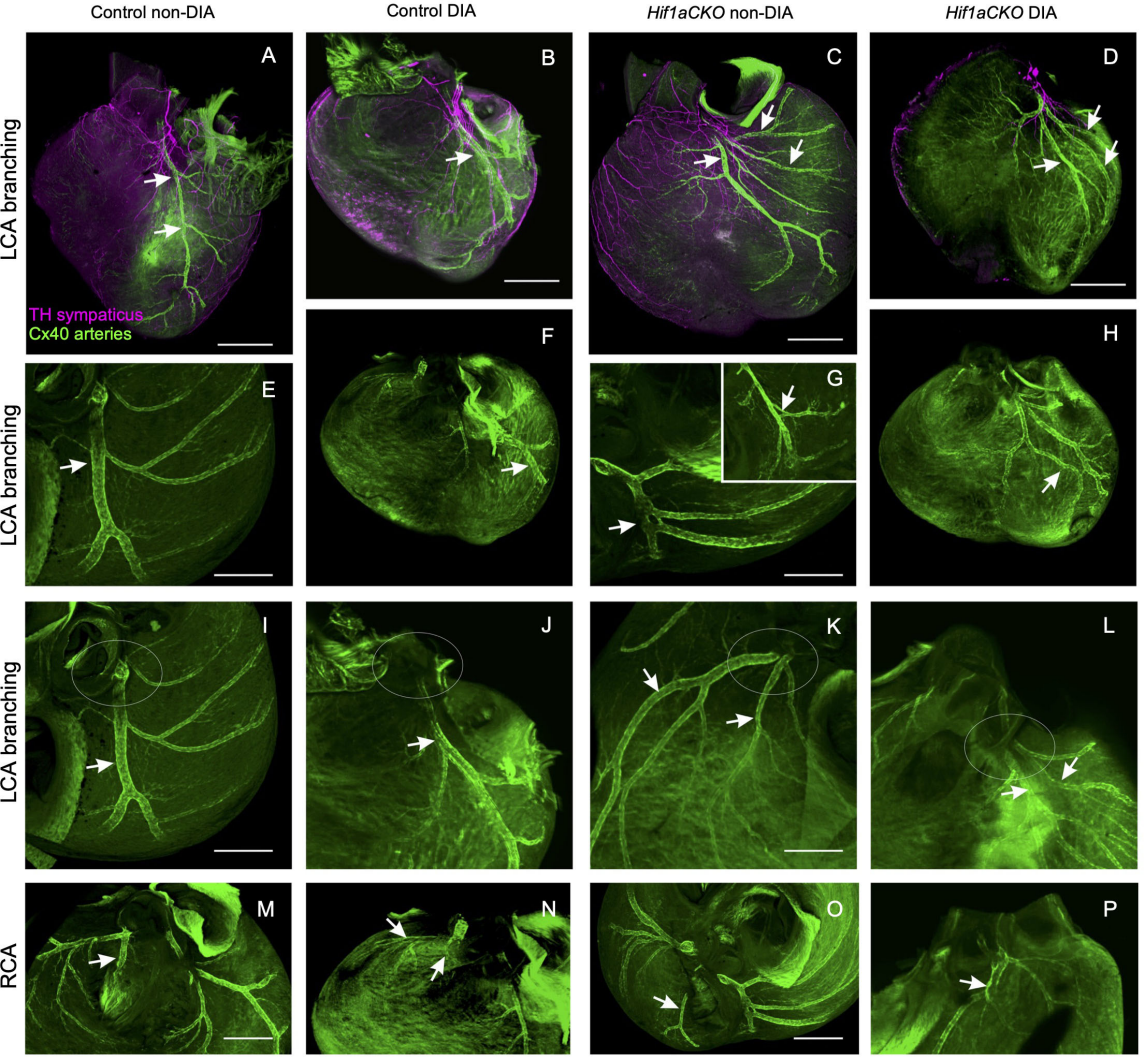
Anomalies of main coronary arteries. **(A)** Normal branching of the left coronary artery (LCA) in control mouse embryo at E17.5 with anterior interventricular branch [left anterior descending (LAD); arrows]. **(B)** Almost normal branching of LCA in embryo of diabetic mother with slightly twisted branches of the LCA. **(C)** Multiple branches of the LCA are radiating to the base of the left ventricle in *Hif1aCKO* embryo. **(D)** An example of abnormal branching of the LCA in diabetic *Hif1aCKO*. Note that the heart is also generally smaller. **(E, F)** More examples of normal shorter and convoluted branches of the LCA (arrow indicates LAD). **(G, H)** Examples of abnormal and doubled branches of the LCA. Arterial windows in *Hif1aCKO* embryos (arrow and inset in **G**). Arrow in panel H shows two parallel branches next to each other with a small anastomosis. **(I, J)** Normal single orifice of LCA from the aorta and two separate openings into the aorta of the anterior interventricular and the circumflex branch of the LCA. The area of the left aortocoronary orifice is outlined by the ovals. **(K, L)** Double opening of the LCA into the aorta. The ovals outline the aortocoronary openings, and the respective branches (LCA and the circumflex branch) are indicated by the arrows. **(M, N)** Normal connection of the septal branch (arrow) of the right coronary artery (RCA). **(O)** Abnormal RCA connection shown in the *Hif1aCKO* heart: the interventricular branch (arrow) is connected directly to the aorta instead of to the RCA. **(P)** Abnormally dilated segment of the RCA (arrow) just before its entry to the aorta. Scale bars, 500 µm **(A–D, F, H)** and 200 µm **(E, G, I–P)**.

Next, we investigated the coronary vascular architecture, as the development of arterial and sympathetic nerve networks is coordinated in terms of both spatial distribution and molecular signaling. Furthermore, our previous study revealed a higher neonatal mortality rate among *Hif1aCKO* mice, which could be partially linked to major coronary artery anomalies (29). Building upon these findings, our current study expands our analyses of the formation of coronary artery architecture, using the *Cx40:eGFP* knock-in model with eGFP signal in the coronary arteries (47). The normal pattern of the coronary arteries together with examples of abnormal findings recorded from the mutant and diabetic hearts is summarized in **Figure 6**. Abnormalities included multiple smaller branches instead of a single large one, arterial “windows”, and anomalous connections to the aorta (separate orifices of the circumflex and left anterior descending branches of the left coronary artery and separate orifice of the septal branch of the right coronary artery) instead of single opening of the left and right coronary arteries. These abnormalities alone cannot account for the approximately 40% perinatal mortality observed in the *Hif1aCKO* mice (29). Thus, the prenatal evaluations did not uncover any new, more severe malformations in the coronary artery architecture in the *Hif1aCKO* embryos, in comparison to the analyses of the adult *Hif1aCKO* heart reported previously (29).

**Figure 7:**
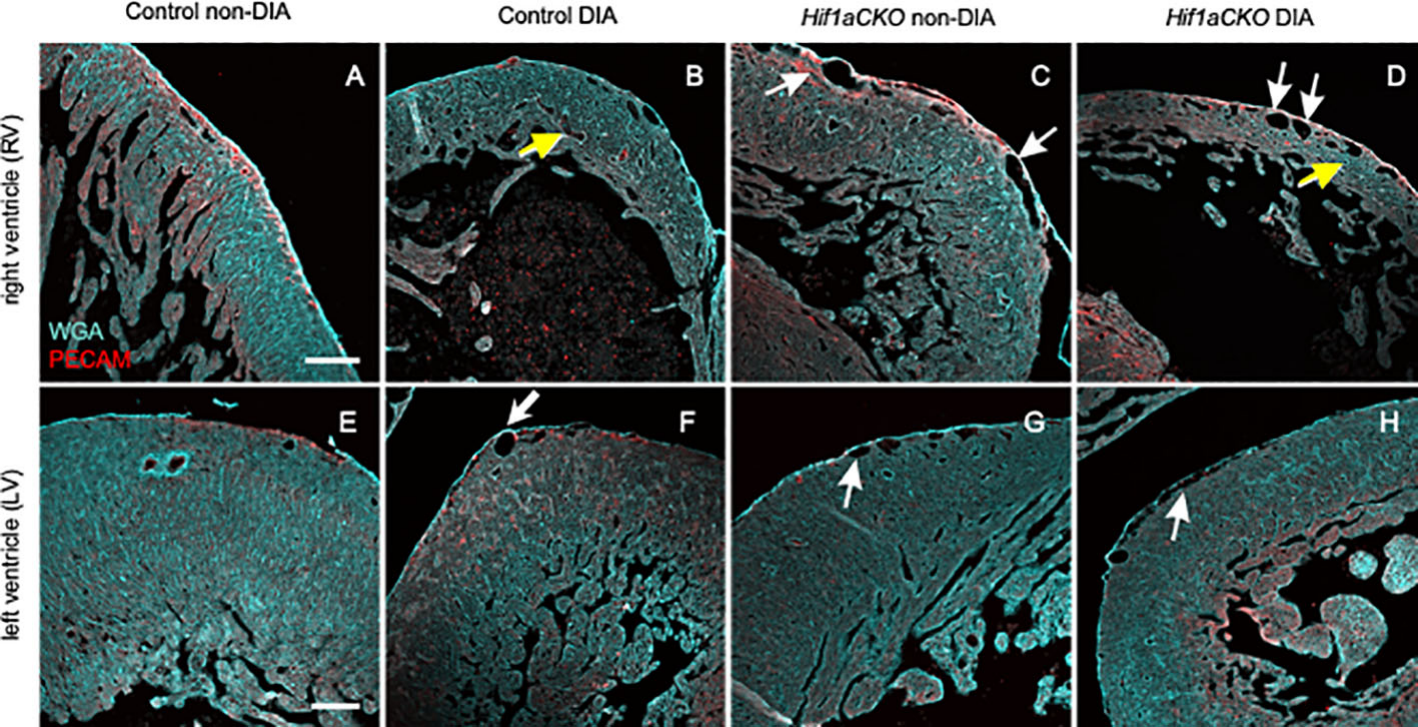
Changes in microvasculature in the embryonal heart induced by maternal diabetes and *Hif1a* deletion. **(A**–**H)** Representative images of visualized microvasculature using a combination of anti-PECAM-1 (a pan-endothelial marker), and wheat germ agglutinin (WGA) labeling depict changes in the E17.5 heart. White arrows indicate dilated subepicardial veins, and yellow arrows indicate round and dilated coronary arteries in the myocardium. Dilated subepicardial veins are observed in the right ventricle (RV) in non-diabetic and diabetic *Hif1aCKO* compared to the control groups. Round and dilated coronary arteries in the myocardium are in the RV of diabetic control and diabetic *Hif1aCKO*. Dilated subepicardial veins in left ventricles (LVs) are found in diabetic control, diabetic, and non-diabetic *Hif1aCKO* groups. Microvasculature in the LV is less affected compared to the RV, although the relative vessel density in the RV is unchanged among the evaluated groups (**Supplementary Figure S3**). Note the thinner compact myocardium of the RV specifically in the diabetic control and *Hif1aCKO* **(B, D)** compared to non-diabetic hearts **(A, C)**. Scale bars, 100 µm.

We next visualized the microvasculature within the heart. We used a combination of anti-PECAM-1 antibody (a pan-endothelial marker) and WGA labeling. Subepicardial veins were noticeably dilated in both non-diabetic and diabetic *Hif1aCKO* in the RV (white arrows in **Figures 7C, D**) when compared to the control hearts from diabetic and non-diabetic embryos (**Figures 7A, B**). Interestingly, coronary arteries in the compact myocardium of the RV appeared dilated in embryos from diabetic pregnancies (yellow arrows in **Figures 7B, D**) but not in non-diabetic *Hif1aCKO* hearts. Although the relative vessel density, indicative of myocardial perfusion and oxygenation in the RV, did not show significant differences among the groups (**Supplementary Figure 3**), the thinner ventricular wall represents a reduction in the absolute amount of microvasculature compared to the controls. The microvasculature of the LV seemed to be less affected, although subepicardial veins were also dilated in diabetic and non-diabetic *Hif1aCKO* and in diabetes-exposed control embryos compared to the non-diabetic control group (**Figures 7E–H**).

### 3.5 HIF-1α deficiency alters the development of adrenal chromaffin cells in diabetic embryos

**Figure 8:**
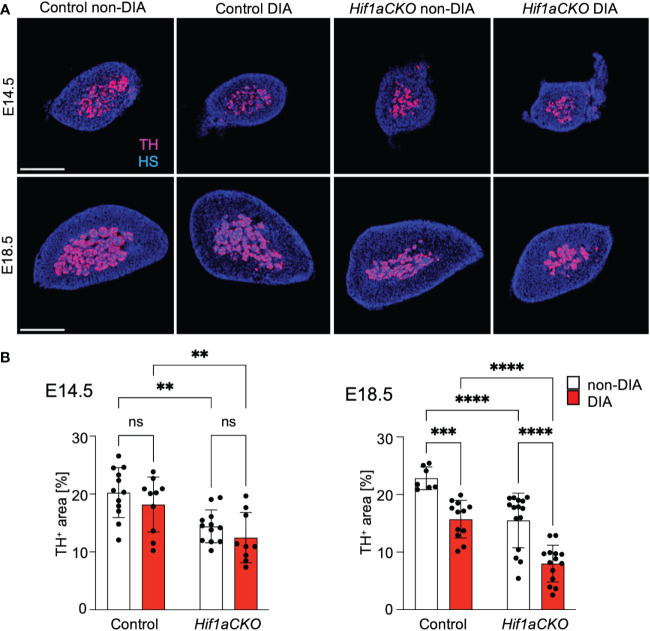
Reduced size of the adrenal medulla in diabetic *Hif1aCKO* mice. **(A)** Representative confocal images of cross-sections through the left adrenal glands of control and *Hif1aCKO*, and diabetic and non-diabetic embryos at E14.5 and E18.5, showing the size of the adrenal medulla by immunolabeled tyrosine hydroxylase (TH), a marker of sympathoadrenal cells. Hoechst-stained cell nuclei (HS). Scale bars, 300 µm. **(B)** TH^+^ areas were quantified using the thresholding tool ImageJ and expressed as a percentage of the total adrenal gland area. The greatest reduction in the size of the adrenal medulla is observed in E18.5 embryos as the result of the combination of *Hif1a* deficiency and maternal diabetes exposure. Data are presented as the mean ± SD (E14.5 n = 12 non-DIA Control, n = 10 DIA Control, n = 12 non-DIA *Hif1aCKO*, and n = 9 DIA *Hif1aCKO*; E18.5 n = 7 non-DIA Control, n = 12 DIA Control, n = 15 non-DIA *Hif1aCKO*, and n = 14 DIA *Hif1aCKO*). Two-way ANOVA followed by *post hoc* Fisher’s multiple comparisons test, **p < 0.01, ***p = 0.001, ****p < 0.0001. ns, not significant.

Sympathetic neurons and neuroendocrine chromaffin cells in the adrenal medulla originate from a common catecholaminergic sympathoadrenal progenitor (62). Therefore, we proceeded to examine the development of chromaffin cells in the adrenal medulla. Consistent with our earlier findings that HIF-1α deficiency adversely impacted the development of chromaffin cells in the adrenal medulla (29), we observed a significant reduction in TH expression in the adrenal medulla *Hif1aCKO* embryos as early as E14.5 (**Figures 8A, B**). However, the detrimental effect of maternal diabetes on TH-expressing chromaffin cells became evident at a later stage, specifically at E18.5. Maternal diabetes exposure led to a significant reduction in the size of the adrenal medulla of control embryos, but diabetic *Hif1aCKO* embryos exhibited the most substantial reduction when compared to the other groups.

### 3.6 *Hif1a* deletion and maternal diabetes impair postganglionic neural development

**Figure 9:**
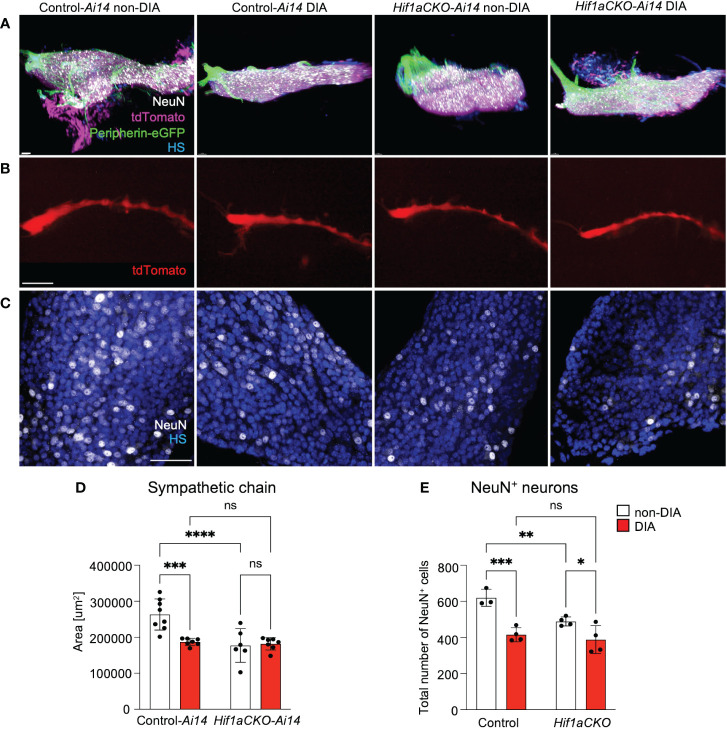
An altered development of sympathetic chain ganglia induced by maternal diabetes and *Hif1a* mutation. **(A)** Representative images of microdissected stellate ganglion (STG) of the secondary sympathetic chain of reporter control-*Ai14* and *Hif1aCKO*-*Ai14* embryos at E14.5. Samples were cleared (CUBIC protocol), imaged, and reconstructed using 3D light-sheet fluorescence microscopy showing tdTomato*^+^* and *Prph*-eGFP^+^ neurons immunolabeled by anti-NeuN (a marker of differentiated neurons). Hoechst-stained cell nuclei (HS). Scale bar, 50 µm. **(B)** Representative images of the secondary sympathetic chain (SG) from STG to fourth thoracic ganglion at E14.5, reconstructed using 3D light-sheet fluorescence microscopy. Scale bar, 500 µm. **(C)** Confocal images of immunostaining for NeuN show neuronal density in the STG ganglion at E14.5. Hoechst-stained cell nuclei (HS). Scale bars, 50 µm. **(D)** Quantification of the area of the sympathetic chain (SG) from STG to fourth thoracic ganglion at E14.5. Data are presented as the mean ± SD (n = 7 non-DIA Control, n = 7 DIA Control, n = 6 non-DIA *Hif1aCKO*, and n = 7 DIA *Hif1aCKO*). **(E)** Density of NeuN^+^ cells was quantified per area of the STG at E14.5. Data are expressed as mean ± SD (n = 3–4 samples per genotype, 3 areas per sample). Two-way ANOVA followed by *post hoc* Fisher’s multiple comparisons test; *p < 0.05, **p < 0.01, ***p < 0.001, ****p < 0.0001. ns, not significant.

Considering the compromised cardiac sympathetic innervation resulting from HIF-1α deficiency and exposure to a maternal diabetic environment, we assessed the development of postganglionic neurons. The stellate ganglia and thoracic sympathetic chain were evaluated using light-sheet fluorescence microscopy and tdTomato and *Prph-*eGFP reporter expression at E14.5 (**Figures 9A, B**). The size of the sympathetic chain was reduced in response to maternal diabetes and *Hif1a* deletion when compared to non-diabetic control sympathetic chains (**Figure 9D**). Similarly, the number of neurons expressing NeuN, a marker of mature neurons, exhibited a decrease in both maternal diabetes and *Hif1a* mutation conditions, with no observed additive combinatorial effect of *Hif1a* mutation within the diabetic environment (**Figures 9C, E**).

**Figure 10:**
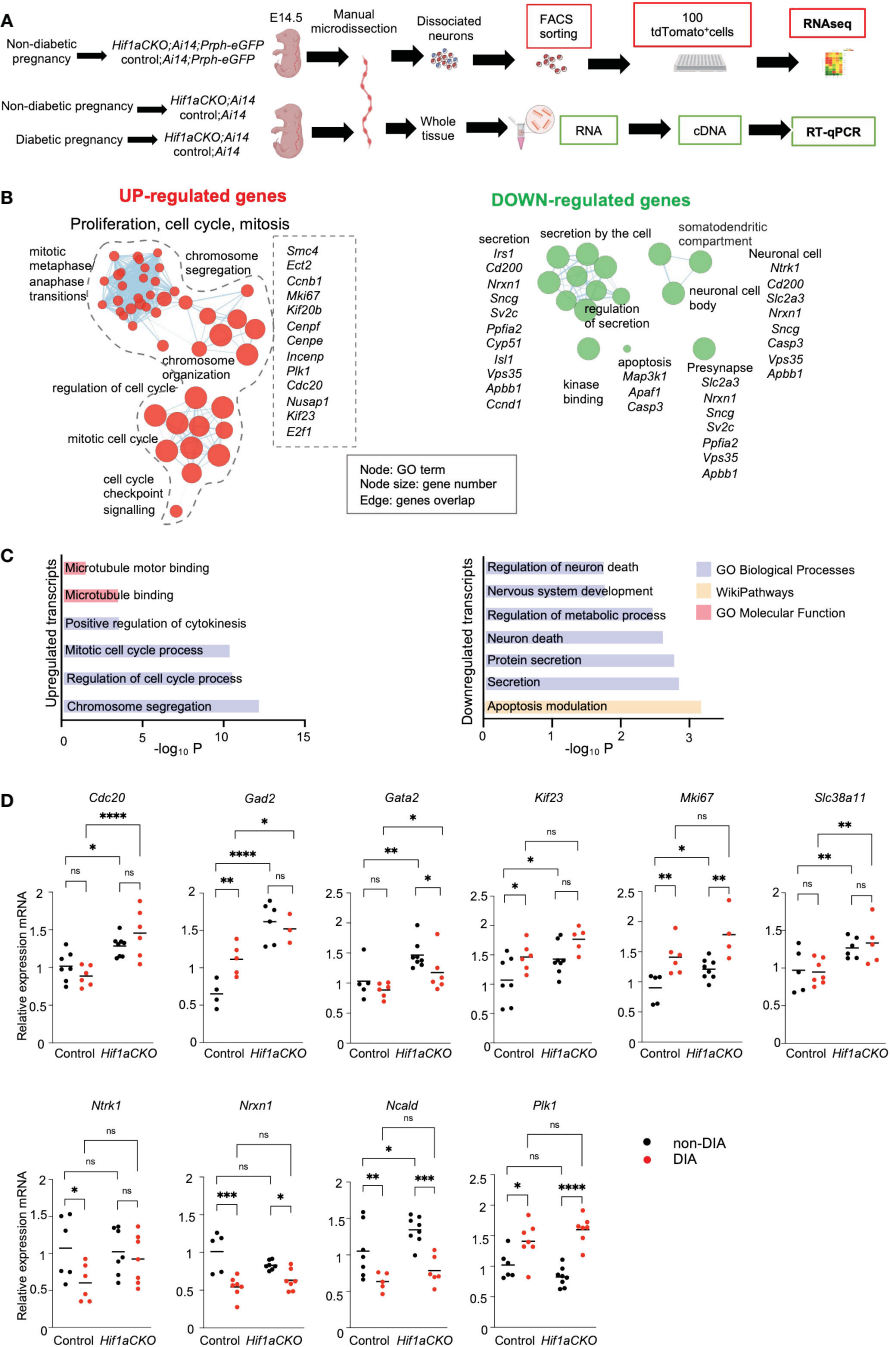
*Hif1a*-mediated transcription signature in sympathetic neurons. **(A)** Workflow depicts microdissection, dissociation, fluorescence-activated cell sorting (FACS) of single tdTomato^+^ and *Prph-*eGFP^+^ sympathetic neurons from control-*Ai14*;*Prph-eGFP* and *Hif1aCKO-Ai14;Prph-eGFP* non-diabetic embryos for a bulk of 100 cell RNA-seq analysis. RT-qPCR analyses were performed using RNA isolated from the microdissected sympathetic chain from E14.5 control and *Hif1aCKO* embryos from diabetic and non-diabetic pregnancies. Created with BioRender.com. **(B)** Gene set enrichment map of downregulated and upregulated differentially expressed genes visualized by the network. The complete list of identified down- and up-differentially expressed genes (adjusted p-value < 0.05, fold change >0.3, and <−0.3 cutoff values) is in **Supplementary Data 1**. Each node represents a Gene Ontology (GO) term; edges are drawn when there are shared genes between two GO terms. Each GO set cluster was assigned representative keywords; a full list of significant GO terms is available in **Supplementary Data 1**. **(C)** The bar graphs demonstrate a significant enrichment of GO terms, which reflect notable changes in sympathetic neurons. **(D)** The expression of RNA-seq-identified differentially expressed genes was analyzed by qRT-PCR. RNA mRNA was isolated from the microdissected secondary sympathetic chain of diabetic and non-diabetic control-*Ai14* and *Hif1aCKO-Ai14* embryos. Two-way ANOVA followed by *post hoc* Fisher’s multiple comparisons test; *p < 0.05, **p < 0.01, ***p < 0.001, ****p < 0.0001, ns, not significant. Abbreviations: *Cdc20*, cell division cycle 20; *Gad2*, glutamic acid decarboxylase 2; *Gata2*, GATA binding protein 2; *Kif23*, kinesin family member 23; *Mki67*, antigen identified by monoclonal antibody Ki 67; *Ncald*, neurocalcin delta; *Ntrk1*, neurotrophic tyrosine kinase receptor, type 1; *Nrxn1*, neurexin I; *Plk1*, polo like kinase 1; *Slc38a11*, solute carrier family 38, member 11.

To analyze the molecular changes resulting from the elimination of *Hif1a* in developing sympathetic neurons, we performed a bulk-cell RNA sequencing (experimental design in **Figure 10A**). Each biological replicate for the bulk RNA-seq analysis contained 100 *Prph-*eGFP^+^ and tdTomato^+^ FACS-sorted single cells from the E14.5 stellate ganglia and thoracic sympathetic chain.

Differential expression analysis identified 36 downregulated and 32 upregulated transcripts of protein-coding genes in *Hif1aCKO* neurons (**Supplementary Data 1**). Functional enrichment analysis of the set of upregulated genes demonstrated a significant enrichment of Gene Ontology (GO) categories associated with *proliferation*, *cell cycle*, and *mitosis*, likely reflecting the main compensatory mechanisms for aberrant neuronal development (**Figures 10B, C**; **Supplementary Data 1**). For example, upregulated genes encoding proteins are involved in cell cycle and mitosis regulation including *Cdc20*, the coactivator of mitotic progression, which is also required for dendrite development (63); the transcription factor *E2f1*, which is important for cell cycle progression and apoptosis (64); cyclin B1 (*Ccnb1*) (65); *Kif23*, which is important for cell division and implicated in neuronal migration (66); *Cenpe*, *Cenpf*, and *Mki67*, which are important for cell cycle and mitosis (67); and *Mcc*, which is a regulator of cell cycle and Wnt/β-catenin signaling pathway (68). Additionally, we detected an elevated expression of glutamic acid decarboxylase 2 (*Gad2*), the enzyme that catalyzes the formation of γ-aminobutyric acid (GABA) from glutamic acid. GABAergic signaling, the main inhibitory neurotransmitter system, is important in sympathetic and cardiovascular regulation (69). *Slc38a11*, a member of the SLC38 family of amino acid transporters, was upregulated, although its function and substrate specificity are unknown (70). Moreover, elevated levels of *Gata2*, a key transcription factor expressed in developing sympathetic neurons (71), were found in *Hif1aCKO* neurons.

The GO analysis of downregulated genes revealed a notable enrichment of biological processes linked to *nervous system development*, *neuron death*, *secretion*, and *synapse function*, indicating significant alternations in neuronal development, survival, and function. The downregulated set included genes such as HIF-1, regulated and highly expressed in neurons glucose transporter (*Slc2a3*) (72), the neuronal calcium sensor neurocalcin delta (*Ncald*) (73), a synaptic organizer and adhesion molecule neurexin 1 (*Nrxn1*) (74), and neurotrophic receptor tyrosine kinase 1, *Ntrk1* (also known as *TrkA*), a receptor for nerve growth factor with a key role in the regulation of proliferation, differentiation, and survival of sympathetic neurons (75, 76).

Using our RNA-seq data, we selected specific genes for qRT-PCR analysis to examine their expression in the sympathetic chain ganglia of control and *Hif1aCKO* embryos from non-diabetic and diabetic pregnancies at E14.5 (experimental design in **Figure 10A**). Consistent with the RNA-seq results, we found significantly increased expression levels of several selected genes, including *Cdc20*, *Gad2*, *Gata2*, *Kif23*, *Mki67*, and *Scl38a11*, in *Hif1aCKO* compared to the control group (**Figure 10D**). Additionally, some of these genes (*Gad2*, *Kif23*, and *Mki67*) were also upregulated in the sympathetic chain ganglia of control embryos exposed to maternal diabetes. While there was no additive effect of maternal diabetes in conjunction with *Hif1a* deletion on their expression, these results suggest possible compensatory mechanisms associated with proliferation, cell cycle, and mitosis in response to the adverse diabetic environment or *Hif1a* deficiency in developing sympathetic neurons. Interestingly, genes associated with synapse function, such as *Nrxn1* and *Ncald*, exhibited reduced expression in both control and *Hif1aCKO* embryos from diabetic pregnancies. In contrast, *Plk1* encoding Polo-like kinase 1, a regulator of mitosis (77), was upregulated by maternal diabetes but not affected by *Hif1a* deletion. Overall, these results align with our analyses of cellular changes in sympathetic chains, with no observed additive combinatorial effect of *Hif1a* mutation in the diabetic environment.

## 4 Discussion

This study investigates the combined impact of the *Hif1a-*deficient sympathetic system and maternal diabetes on heart development, specifically focusing on cardiac innervation, coronary artery formation, and the development of postganglionic sympathetic neurons of the cardiac sympathetic system. It provides the first comprehensive analysis of how maternal diabetes exposure affects the development of the cardiac sympathetic system, highlighting the combined impact of the *Hif1a-*deficient sympathetic system and the maternal diabetes environment on the heart. Inadequate activation of the HIF-1α regulatory pathway, particularly in the context of maternal diabetes, may contribute to abnormalities in the cardiac sympathetic system.

Diabetic pregnancies are associated with an increased incidence of congenital anomalies (78, 79) and an increased risk of fetal and infant death (80). The risk of fetal death is over four times higher, and the risk of infant death is nearly doubled in diabetic pregnancies (80). Furthermore, both human and animal studies indicate that exposure to diabetes *in utero* increases cardiovascular risk factors in the offspring, with long-term consequences for cardiovascular and metabolic health (37, 79, 81–83). Given the known associations between sympathetic defects and conditions like sudden infant death syndrome, cardiac arrhythmic death, and certain congenital heart defects in children (23, 24), we hypothesize that the *Hif1a* deficiency combined with maternal diabetes may further compromise the development of the cardiac sympathetic system. This compromise could potentially contribute to cardiac abnormalities and heightened health risks.

Consistent with our previous research (29), we observed a significant deficiency in cardiac sympathetic innervation and the development of neuroendocrine chromaffin cells in *Hif1aCKO* mice. Notably, the combination of the *Hif1a*-deficient sympathetic system and exposure to maternal diabetes accelerated the impairment of sympathetic innervation in the developing heart. Additionally, the negative impact on the size of the adrenal medulla in *Hif1aCKO* was further amplified by maternal diabetes, indicating an additive effect of *Hif1a* deletion and diabetes exposure. Chromaffin cells of the adrenal medulla are an important component of the sympathetic system and modulators of metabolic stress responses (84).

Previously, we reported a 40% increased neonatal mortality rate among *Hif1aCKO* mice (29). A similar reduced survival was also reported for mice with germline deletion of *Th*, although the cause of death in these mice was undetermined (1). In the current study, considering the coordinated development of peripheral sympathetic innervation and the coronary arterial network, we investigated the presence of anomalies in the coronary vasculature. We used genetically labeled coronary arteries in the *Cx40:eGFP* knock-in model (47) to examine coronary vascular development. While we observed abnormalities in the architecture of coronary arteries of *Hif1aCKO* embryos, they alone cannot account for the 40% neonatal mortality in *Hif1aCKO*.

Given the implication of the sympathetic nervous system in heart size regulation and cardiomyocyte proliferation (19, 20), we compared the size of the heart and the compact myocardial wall thickness. Exposure to maternal diabetes and *Hif1a* mutation had adverse effects on both heart size and compact myocardium of the LV and RV. We did not detect an additive effect of the combination of the *Hif1a-*deficient sympathetic system and the diabetic environment on these parameters compared to diabetic control embryos. The reduction in ventricular wall thickness observed in mutant and diabetes-exposed embryos, in comparison to non-diabetic controls, implies a corresponding decrease in the absolute amount of microvasculature. Consequently, this reduction is anticipated to impact myocardial perfusion and oxygenation. Moreover, dilated subepicardial veins and coronary arteries in the myocardium in both diabetic and non-diabetic *Hif1aCKO*, as well as diabetic control embryos, indicate compromised cardiac function. Increased thickness of the interventricular septum in these hearts suggests a compensatory response to failing hearts, given the smaller heart size and thinner ventricular walls. Whether this is a secondary effect or a direct result of the deficient adrenergic innervation remains unclear.

Our assessment of the cellular and molecular changes in developing postganglionic sympathetic neurons revealed significant alternations induced by *Hif1a* deletion or maternal diabetes at E14.5. However, we did not observe an additive combinatorial effect of *Hif1a* mutation in the diabetic environment. We selected the E14.5 developmental stage of sympathetic neurons for two main reasons. First, E14.5 represents a peak exit from the cell cycle, although ∼25% of neurons are still cycling at E18.5 (8). Second, the first axons reach the heart ∼E14 (17); therefore, we hypothesize that molecular changes associated with aberrant axonogenesis of sympathetic neurons might be detectable by RNA-seq at this stage. We found a significant enrichment of upregulated genes associated with proliferation, cell cycle, and mitosis, reflecting the main compensatory mechanisms for aberrant neuronal development of *Hif1aCKO*. In contrast, downregulated genes in *Hif1aCKO* were associated with nervous system development, synaptic function, and neuronal death, indicating altered neuronal development.

It is important to acknowledge the limitations of our study design. By focusing our molecular analysis (RNA-seq) on a single time point, we obtained only limited information about the temporal aspects of these molecular changes in *Hif1aCKO* sympathetic neurons. Another limitation of this study is that bulk-cell RNA sequencing approaches provide an average of expressional differences from multiple neurons, obscuring cell-specific differences. To address these limitations, future investigations using single-cell RNA-seq will be needed to fully establish molecular differences linked to specific cell states, cell-to-cell variability, to uncover the pathways of cell lineage differentiation affected in sympathetic neurons.

In summary, our results indicate that the interplay between deficiencies in the sympathetic system and subtle structural alternations in the vasculature, microvasculature, myocardium, and septum during heart development increases the risk of fetal demise for *Hif1aCKO*. Furthermore, even normal coronary vasculature, when experiencing deficient innervation, may not adequately react to the stress associated with birth and the subsequent rapid adaptation to oxygen breathing. Specifically, insufficient vasodilatation *via* beta-adrenergic receptors could lead to compromised cardiac function and neonatal death. This hypothesis is supported by considerable variability in the degree of sympathetic innervation in *Hif1aCKO*; it is likely that those with the most severe deficit are among the 40% of neonatal deaths. Considering the rapid clearance of dead pups by the mother, this hypothesis was difficult to validate. Deficient sympathetic innervation was reported from failing human hearts (85) or animal models of heart failure (86). Both deficient sympathetic innervation of the fetal heart along with the hypoplastic adrenal medulla affecting circulating catecholamine levels found in our study can contribute to this cascade of adverse peripartum events.

## Data availability statement

The RNAseq data presented in the study are deposited in the GEO depository, accession number GSE250606.

## Ethics statement

The animal study was approved by the Animal Care and Use Committee of the Institute of Molecular Genetics, CAS, and First Faculty of Medicine, CUNI. The study was conducted in accordance with the local legislation and institutional requirements.

## Author contributions

HK: Formal analysis, Investigation, Methodology, Validation, Visualization, Writing – review & editing. PH: Formal analysis, Funding acquisition, Investigation, Methodology, Validation, Visualization, Writing – original draft. RB: Data curation, Formal analysis, Investigation, Methodology, Supervision, Validation, Writing – review & editing. PA: Investigation, Methodology, Writing – review & editing. VF: Investigation, Methodology, Visualization, Writing – review & editing. DS: Conceptualization, Formal analysis, Investigation, Methodology, Supervision, Validation, Visualization, Writing – review & editing. GP: Conceptualization, Funding acquisition, Project administration, Supervision, Writing – original draft, Writing – review & editing.

## Supplementary material

Supplementary Video S1 Non-diabetic control embryonic heart at E16.5. Microdissected embryonic heart was cleared (CUBIC protocol), imaged, and reconstructed in 3D using light-sheet fluorescence microscopy (LFSM). Video shows the cardiac innervation using immunohistochemical staining of the sympathetic neuron marker, tyrosine hydroxylase (TH), and TUJ1 as a neuron marker.

Supplementary Video S2 Diabetic control embryonic heart at E16.5. Microdissected embryonic heart was cleared (CUBIC protocol), imaged, and reconstructed in 3D using light-sheet fluorescence microscopy (LFSM). Video shows the cardiac innervation using immunohistochemical staining of the sympathetic neuron marker, tyrosine hydroxylase (TH), and TUJ1 as a neuron marker.

Supplementary Video S3 Non-diabetic Hif1aCKO embryonic heart at E16.5. Microdissected embryonic heart was cleared (CUBIC protocol), imaged, and reconstructed in 3D using light-sheet fluorescence microscopy (LFSM). Video shows the cardiac innervation using immunohistochemical staining of the sympathetic neuron marker, tyrosine hydroxylase (TH), and TUJ1 as a neuron marker.

Supplementary Video S4 Diabetic Hif1aCKO embryonic heart at E16.5. Microdissected embryonic heart was cleared (CUBIC protocol), imaged, and reconstructed in 3D using light-sheet fluorescence microscopy (LFSM). Video shows the cardiac innervation using immunohistochemical staining of the sympathetic neuron marker, tyrosine hydroxylase (TH), and TUJ1 as a neuron marker.
