# Supplementary material for: Reprogramming of the developing heart by Hif1a-deficient sympathetic system and maternal diabetes exposure
Source: Front Endocrinol (Lausanne). 2024 Mar 5;15:1344074. doi: 10.3389/fendo.2024.1344074 (PMC10948485; doi:10.3389/fendo.2024.1344074)
Supplement: Supplementary file 6 [file Presentation_1.pdf]

Supplementary Material

1 Supplementary Tables and Figures

1.1 Supplementary Tables 1 - 4

1.2 Supplementary Figures 1 - 3

Supplementary Table S1: Primer sequences for genotyping

Gene symbol	Forward primer sequence	Reverse primer sequence
Hif1a flox	5'-TGCATGTGTATGGGTGTTTTG-3'	5'-GAAAACTGTCTGTAACTTCATTTCC-3'
Isl1/Cre	5’-GCCTGCATTACCGGTCGATGCAACGA-3'	5’-GTGGCAGATGGCGCGGCAACACCATT-3'
WT- TomatoAi14	5´-AAGGGAGCTGCAGTGGAGTA-3´	5´-CCGAAAATCTGTGGGAAGTC-3´
Transgene-TomatoAi14	5´-CTGTTCCTGTACGGCATGG-3´	5´-GGCATTAAAGCAGCGTATCC-3´
eGFP	5′-GCACGACTTCTTCAAGTCCGCCATGCC-3′	5′-GCGGATCTTGAA GTTCACCTTGATGCC-3´
Cx40	5′-CTCCAATTAACTCCTTGTGAGCC-3′	5′-AGGCTGAATGGTATCGCACC-3′
Neo for e*GFP* insert in Cx40:eGFP genotyping		5′-CTTGCCGAATATCATGGTGG-3′

Supplementary Table 2: Primary antibodies

**Primary antibody against**	**Host species**	**Company**	**Catalog number**	**IHC dilution**
NeuN	rabbit	Abcam	ab177487	1:500
PECAM-1	mouse	Santa-Cruz Biotech	sc-376764	1:200
TH	rabbit	Merck Millipore	ab152	1:500
TUJ1	mouse	BioLegend	801202	1:500
WGA coupled with Alexa fluor 488 dye		Invitrogen	W11261	1:50

Abbreviations:; NeuN, neuronal nucleus marker; PECAM, platelet endothelial cell adhesion molecule; TH, tyrosine hydroxylase; TUJ1, neuron-specific class III beta-tubulin; WGA, Wheat Germ Agglutinin.

Supplementary Table 3: Secondary antibodies

**Secondary antibody**	**Company**	***Catalog number***	**Dilution**
Alexa Fluor® **488-**conjugated AffiniPure Donkey Anti-**Mouse** IgG (H+L)	Jackson ImmunoResearch	715-545-150	1:500
Alexa Fluor® **594**-conjugated AffiniPure Donkey Anti-**Rabbit** IgG (H+L)	Jackson ImmunoResearch	711-585-152	1:500
Cy™5 AffiniPure Goat Anti-**Mouse** IgG (H+L)	Jackson ImmunoResearch	115-175-146	1:200
Cy™5 AffiniPure Goat Anti-**Rabbit** IgG (H+L)	Jackson ImmunoResearch	111-174-144	1:200
Alexa Fluor® **647**-conjugated AffiniPure Donkey Anti-**Rabbit** IgG (H+L)	Jackson ImmunoResearch	715-605-152	1:500

Supplementary Table 4: Primer sequences for quantitative real-time polymerase chain reaction

**Gene symbol**	**Forward primer sequence**	**Reverse primer sequence**
Cdc20	5'-GGCTCCCCTGCAAACATTCA-3'	5'-TGAGCAGACGTTCCAAATGC-3'
Gad2	5'-GAGGGCTCCTGTGGCAAAT-3'	5'-TGGAACCACCGTGTATGGGG-3'
Gata2	5'-CCCAAGCTTCGATTCTGTGT-3'	5'-TTGACTCAGCACAATCGTCTC-3'
Hprt1	5'-GCTTGCTGGTGAAAAGGACCTCTCGAAG-3'	5'-CCCTGAAGTACTCATTATAGTCAAGGGCAT-3'
Kif23	5'-TGCCAACTGGTAGTCGGAAA-3'	5'-TTTCTTGCGCTTGGGTTGTG-3'
Mki67	5'-AACCATCATTGACCGCTCCTT-3'	5'-TTGACCTTCCCCATCAGGGT-3'
Ncald	5'-CCCCACTTCTTCAGCGTGAG-3'	5'-ACTCCTGGATCTCGTGCTCT-3'
Nrxn1	5'-GACAACTGAGTCGACAGCCA-3'	5'-GGCTCACAGGGGTCAATGTC-3'
Ntrk1	5'-GCCTAACCATCGTGAAGAGTG-3'	5'-CCAACGCATTGGAGGACAGAT-3'
Plk1	5'-CACGTCGTAGGCTTCCATGA-3'	5'-GAATGACCTGATTGCGGTGC-3'
Slc38a1	5'-CAAAGACTCCCAGGAGTTGACCC-3'	5'-GGCCCTTGTCATTACGATTCCA-3'

*Primers were designed using Primer3 and BLAST software. Abbreviations: *Cdc20*, cell division cycle20 ; *Gad2*, glutamic acid decarboxylase 2; *Gata2*, GATA binding protein 2; *Hprt1*, hypoxanthine guanine phosphoribosyl transferase; *Kif23* kinesin family member 33; *Mki67*, marker of proliferation Ki67; *Ncald*, neurocalcin delta; *Nrxn1*, neurexin; *Ntrk1,* neurotrophic tyrosine kinase receptor type 1; *Plk1*, polo like kinase 1; *Slc38a1*, solute carrier family 38 member 11.

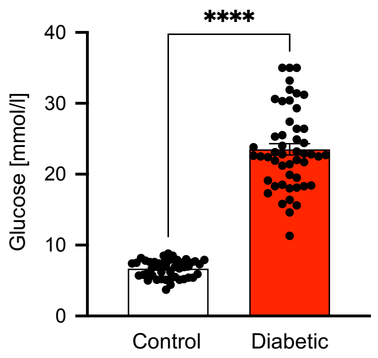

Supplementary Figure 1. Maternal blood glucose levels at time of embryo collections. Data are presented as mean ± SEM (n = 46 control, n = 50 diabetic). Unpaired two-tailed *t*-test, **** $P < 0.0001$.

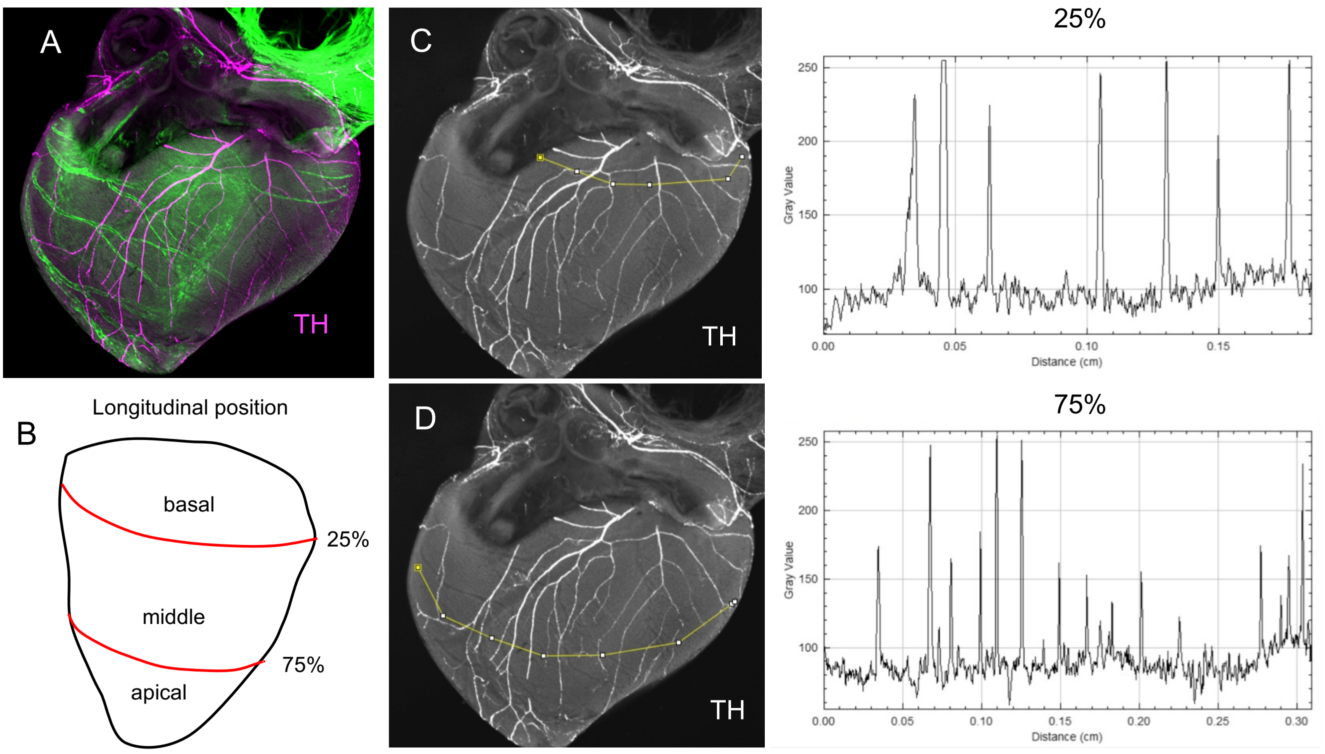

Supplementary Figure 2. Innervation evaluation method. (**A**) Representative image of immunolabeling of sympathetic innervation in the E17.5 heart labeled by anti-tyrosine hydroxylase (TH). (**B**) Schematics of the heart oriented with the base on the top and apex at the bottom (Valentine projection). Red lines indicate positions for 25% (basal) and 75% (apical) apex-base distance for semicircular lines to generate a profile of fluorescence intensity corresponding individual nerve fibers using ImageJ. (**C, D**) Examples of the evaluated image with drawn semicircular segmented lines (yellow) 25% basal and 75% apical with corresponding histograms.

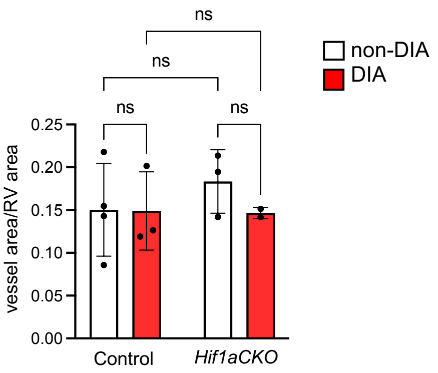

Supplementary Figure 3. The evaluation of the relative density of microvasculature in the right ventricle of the embryonic heart at E17.5. Data are presented as mean ± SD. Two-way ANOVA followed by post hoc Fisher‘s multiple comparisons test ns, non-significant
